# Cortactin Is a Substrate of Activated Cdc42-Associated Kinase 1 (ACK1) during Ligand-induced Epidermal Growth Factor Receptor Downregulation

**DOI:** 10.1371/journal.pone.0044363

**Published:** 2012-08-30

**Authors:** Laura C. Kelley, Scott A. Weed

**Affiliations:** Department of Neurobiology and Anatomy, Program in Cancer Cell Biology, Mary Babb Randolph Cancer Center, West Virginia University, Morgantown, West Virginia, United States of America; University of Birmingham, United Kingdom

## Abstract

**Background:**

Epidermal growth factor receptor (EGFR) internalization following ligand binding controls EGFR downstream pathway signaling activity. Internalized EGFR is poly-ubiquitinated by Cbl to promote lysosome-mediated degradation and signal downregulation. ACK1 is a non-receptor tyrosine kinase that interacts with ubiquitinated EGFR to facilitate EGFR degradation. Dynamic reorganization of the cortical actin cytoskeleton controlled by the actin related protein (Arp)2/3 complex is important in regulating EGFR endocytosis and vesicle trafficking. How ACK1-mediated EGFR internalization cooperates with Arp2/3-based actin dynamics during EGFR downregulation is unclear.

**Methodology/Principal Findings:**

Here we show that ACK1 directly binds and phosphorylates the Arp2/3 regulatory protein cortactin, potentially providing a direct link to Arp2/3-based actin dynamics during EGFR degradation. Co-immunoprecipitation analysis indicates that the cortactin SH3 domain is responsible for binding to ACK1. In vitro kinase assays demonstrate that ACK1 phosphorylates cortactin on key tyrosine residues that create docking sites for adaptor proteins responsible for enhancing Arp2/3 nucleation. Analysis with phosphorylation-specific antibodies determined that EGFR-induced cortactin tyrosine phosphorylation is diminished coincident with EGFR degradation, whereas ERK1/2 cortactin phosphorylation utilized in promoting activation of the Arp2/3 regulator N-WASp is sustained during EGFR downregulation. Cortactin and ACK1 localize to internalized vesicles containing EGF bound to EGFR visualized by confocal microscopy. RNA interference and rescue studies indicate that ACK1 and the cortactin SH3 domain are essential for ligand-mediated EGFR internalization.

**Conclusions/Significance:**

Cortactin is a direct binding partner and novel substrate of ACK1. Tyrosine phosphorylation of cortactin by ACK1 creates an additional means to amplify Arp2/3 dynamics through N-WASp activation, potentially contributing to the overall necessary tensile and/or propulsive forces utilized during EGFR endocytic internalization and trafficking involved in receptor degradation.

## Introduction

Ligand-induced endocytic regulation of EGFR trafficking is utilized as a key mechanism for modulating growth factor signaling by controlling levels of EGFR surface expression [1]. Ligand-bound, dimerized EGFR is rapidly internalized by clathrin-mediated endocytosis (CME), where it traffics to early endosomes and is segregated to either multivesicular endosomes (or “bodies”; MVBs) for lysosomal-mediated degradation, or sorted to recycling endosomes and reinserted into the plasma membrane [2]. Endocytic internalization of activated EGFR is regulated by multiple interacting factors, including receptor ubiquitination by Cbl, binding to the adaptor proteins Grb2 and AP-2, and receptor acetylation [3], [4], [5]. In addition to CME, EGFR can be internalized by recruitment into cholesterol-rich caveolae [6], macropinocytosis [7] or by circular dorsal ruffles generated from cortical actin polymerization [8]. These CME-independent mechanisms are typically employed by cells either exposed to high experimental EGF concentrations or in cells with increased levels of surface EGFR expression [1], [2]. EGFR overexpression is a frequent occurrence in many cancers that corresponds to poor clinical outcome, including breast [9] and head and neck squamous cell carcinoma (HNSCC) [10].

Activation of EGFR is requisite for receptor internalization, triggering downstream activation of additional non-receptor tyrosine kinases that participate in the internalization process [11], [12]. ACK1 is a multidomain non-receptor tyrosine kinase that contributes to EGFR internalization and subsequent vesicular trafficking (reviewed in [13]). EGF stimulation recruits ACK1 to activated EGFR through an ACK1 carboxyl-terminal region highly homologous to the EGFR binding protein Mig6, which in turn activates ACK1 kinase activity [14], [15]. ACK1 also interacts with clathrin heavy chain [16], [17] and Grb2 [14], providing additional attachment points to the endocytic machinery. While ACK1 is well positioned to govern EGFR CME, studies manipulating ACK1 expression levels to evaluate the impact of ACK1 on EGFR internalization have yielded opposing results. In some cases ACK1 overexpression impairs and knockdown promotes EGFR internalization [18], [19]. These findings are in agreement with studies on transferrin receptor CME with the ACK1 splice variant ACK2 [20]. Other work indicates that ACK1 knockdown suppresses EGFR internalization and degradation [15]. This effect was verified by expression of the ACK1 Mig6 domain and with an ACK1 ubiquitin-binding deficient mutant [21], [22]. Like EGFR, in some instances ACK1 itself is downregulated following EGF stimulation due to Nedd4 ubiquitination, targeting ACK1 for either conventional proteosomal [21] or lysosomal [23] degradation depending on the specific Nedd4 isoform. Nedd4-1 ubiquitination of ACK1 is required for EGFR degradation, indicating that at least a subset of EGFR/ACK1 complexes are targeted for vesicular-mediated co-destruction. Supporting these biochemical findings, activated ACK1 is enriched in clathrin-coated pits and colocalizes EGFR in early endosomal vesicles following EGF stimulation [15], [17], [18], suggesting that ACK1 activity regulates EGFR vesicle trafficking in the clathrin pathway.

In addition to collective tyrosine kinase activity at clathrin coated pits, receptor internalization by CME involves coordinate regulation of the cortical actin cytoskeleton by actin regulatory proteins (reviewed in [24]). Activation of Arp2/3 complex, a major actin nucleation apparatus, results in dynamic branched actin network formation at the site of clathrin coated pits that contributes to vesicle invagination, scission and intracytoplasmic transport [25]. Arp2/3 complex activity is stimulated by proteins termed nucleation promoting factors (NPFs) [26]. The type I NPFs of the WASp protein family N-WASp and WASH play important roles in governing Arp2/3 actin polymerization that drives CME and vesicle trafficking [27]. In addition to type I NPFs, the type II NPF cortactin plays a direct role in regulating CME and vesicle motility [28], [29], [30]. Cortactin links the Arp2/3 F-actin network to CME through binding of its carboxyl terminal SH3 domain to the large GTPase dynamin2 [31], [32]. Cortactin and dynamin2 are both tyrosine phosphorylated by Src, events that are required for each protein to function during CME [33], [34]. In the case of cortactin, tyrosine phosphorylation by Src or Abl-family kinases occurs on three tyrosine residues (Y421, 466 and 482 in rodent forms; Y421, 470 and 486 in human) that create docking sites for the adaptor Nck1, which in turn complexes with N-WASp and WIP to amplify Arp2/3 nucleation and enhance actin polymerization levels [35], [36]. In addition to promoting actin nucleation during endocytosis, the cortactin SH3 domain also binds Hip1R to suppress actin dynamics during CME [37], indicating that cortactin serves as an important intersection point in regulating endocytic actin polymerization by phosphorylation -independent and -dependent processes.

EGFR internalization and lysosomal degradation relies in part on direct interactions between the activated receptor and F-actin [38]. Additionally, cortactin modulates EGFR internalization through actin-based interactions with the adaptor protein CD2AP in circular dorsal ruffles, which also require cortactin tyrosine phosphorylation to form [36], [39]. While these studies did not evaluate EGFR degradation, subsequent work in tumor cells with amplified cortactin gene levels indicates that cortactin upregulation prevents EGFR degradation [40], although the mechanism for this is unclear. Here we report that cortactin interacts with ACK1 to potentially regulate EGFR degradation. Cortactin is directly phosphorylated by ACK1 and both proteins localize in vesicles with ligand bound EGFR. Downregulation of cortactin tyrosine phosphorylation is coincident with EGFR degradation, while knockdown of ACK1 or cortactin attenuates EGFR dowregulation through the cortactin SH3 domain. Our results provide evidence for a novel linkage between ACK1 and cortactin that may play an important role in coupling EGFR to the actin cytoskeleton to facilitate EGFR degradation.

## Materials and Methods

### Cell Culture and Transfection

COS1 and 293T cells (purchased from the American Type Culture Collection) were maintained in DMEM (Mediatech) containing 10% FBS (HyClone), 1% L-glutamine and 1% penicillin-streptomycin. 1483 and 584 HNSCC cell lines [41], [42] were maintained as described [43]. COS1 cells (1×10^6^) were transfected with 5 micrograms of plasmid DNA using SuperFect (Qiagen). 293T, 1483 and 584 cells (3×10^6^) were transfected with 2 micrograms of plasmid DNA or siRNA using the Nucleofector I device (Amaxa Biosystems). Control and human targeted siRNA for ACK1 (5′-AAGGUCAGCAGCACCCACUAU-3′) were purchased from Thermo Scientific. Cortactin siRNA (catalog # SI00300160) was purchased from Qiagen.

**Figure 1:**
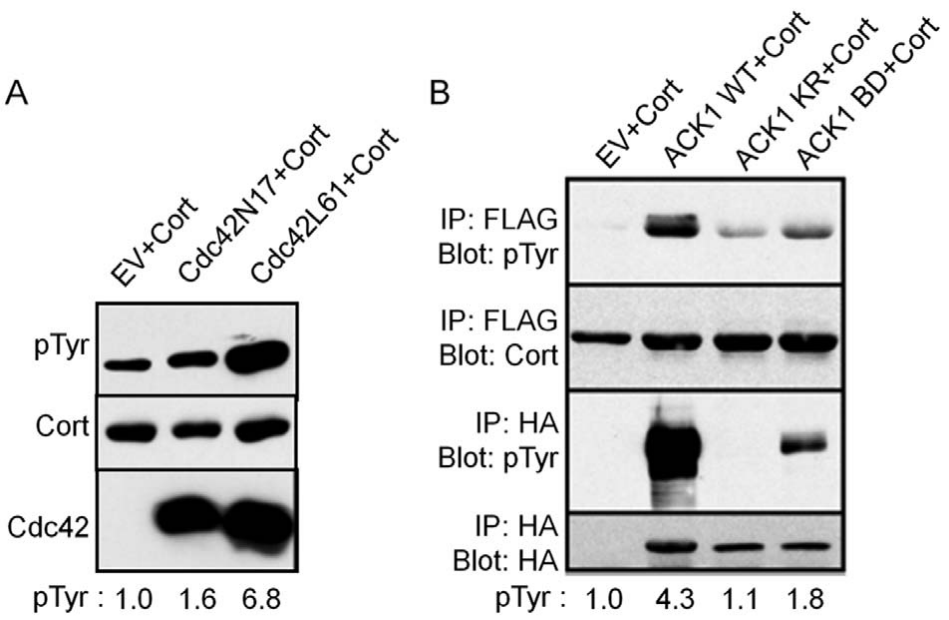
ACK1 mediates Cdc42-induced cortactin tyrosine phosphorylation. (A) COS1 cells were cotransfected with FLAG-cortactin WT and Myc-tagged Cdc42 constructs as indicated. EV; empty vector. Cells were lysed 18 h after transfection, FLAG-cortactin immunoprecipitated and total cortactin tyrosine phosphorylation analyzed by Western blotting with a pan anti-phosphotyrosine antibody (pTyr). The blot was then stripped and reprobed for total cortactin levels (Cort). Equal amounts of total cell lysates were blotted in parallel with anti-Cdc42 antibodies to confirm GTPase expression. Ratios of tyrosine phosphorylated (pTyr) cortactin to total cortactin levels are indicated at the bottom. Blots are representative from ≥ three independent experiments. (B) ACK1 overexpression induces cortactin tyrosine phosphorylation. COS1 cells transfected with empty HA vector (EV), HA-ACK1 wild-type (WT), kinase dead (KR) (K-R mutation at amino acid 158 in the kinase domain) and a Cdc42 binding-null mutant (BD) (H-A mutation of codons 464 and 467 in the CRIB domain) were immunoprecipitated from lysates with anti-FLAG or anti-HA antibodies. FLAG-cortactin and HA-ACK1 proteins were evaluated for phosphotyrosine levels by Western blotting (pTyr). Expression levels for each protein were evaluated by stripping and reblotting with anti-cortactin and anti-HA antibodies as indicated. Ratios of tyrosine phosphorylated (pTyr) cortactin to total cortactin levels are indicated at the bottom. Blots are representative from three independent experiments.

### Cloning of Murine ACK1

Murine ACK1 (GenBank # AF037260) was cloned using two sets of degenerate oligonucleotide primers (primer 1: 5′-GA(T/C)ACNTGGATGTT(T/C)GGNGT-3′; primer 2: 5′-TGGGA(A/G)ATGTT(T/C)ACNTA(T/C)GG-3″; primer 3: 5′-GGNGG(A/G)TCCATNGG(A/G)TTNCC-3′ and primer 4: 5′;TCNGG(A/G)AANCCCCA(A/G)CA(A/G)TG-3′. These primers were used in nested PCRs to generate a 450 bp probe from a mouse brain cDNA library (Novagen). This probe was labeled with ^32^P-dCTP using the RadPrime DNA labeling system (Invitrogen) and used to screen a mouse brain lambda GT10 cDNA library (Stratagene). Positive overlapping clones were identified after three rounds of screening with high stringency conditions. The reconstructed ACK1 sequence consisted of a 1,002 amino acid open reading frame with a polyadenylation tail, but lacked a Kozak consensus sequence with an initiation methionine. The 5′ ACK1 sequence was isolated using a two-step nested PCR approach. The first step used the ACK1 specific primer 5′-ACTGGCATGGGAGAAAGTCCG-3′ with a T7 terminator primer homologous to the vector sequence. The product from this reaction was used as the template for the second reaction using the ACK1 specific primer 5′-TGTTCTTGCATGACATAGTGG-3′ with a primer homologous to the SP6 vector sequence. This reaction generated a 1 kb PCR product containing >600 bp overlap with the original isolated ACK1 cDNA. A Kozak sequence and initiation methionine were identified 53 amino acids upstream from the 5′-end of the original ACK1 clone. Additional PCR was conducted to generate the full-length murine ACK1 coding sequence.

### Mammalian Expression Constructs

For generating HA-tagged ACK1 variants, full-length ACK1 cDNA was excised from pBluescript SK+ as a BamHI/EcoRV fragment and subcloned into BamHI/EcoRV digested pSV containing a 5′ HA epitope tag. The kinase null K158R (KR) and Cdc42-binding null H464/H467Q (BD) ACK1 variants were produced by site directed mutagenesis (QuickChange; Stratagene). Myc-tagged ACK1 constructs were produced by BamHI/EcoRI digestion of pSV-HA ACK1 constructs and subcloning the ACK1 fragments into pRK5Myc [44]. Myc-Cdc42, Myc-RhoA, FLAG-cortactin and CMV-Src527F constructs have been described previously [44], [45], [46], [47].

**Figure 2:**
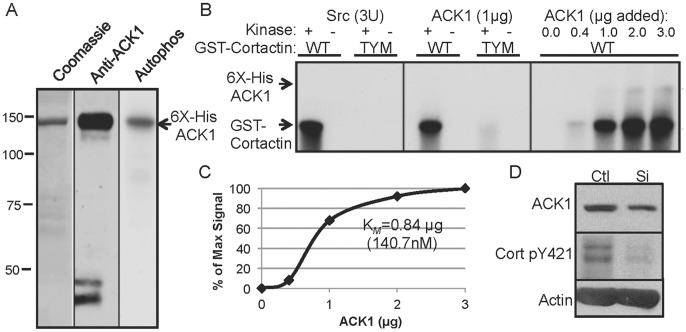
ACK1 directly phosphorylates cortactin at Y421/466/482. (A) Characterization of purified ACK1. Recombinant 6X-histidine tagged recombinant ACK1 was purified from SF9 cells and 5 micrograms stained with either Coomassie blue (*left*) or analyzed by Western blotting with anti-ACK antibodies (*middle*). Kinase activity was assayed by analyzing autophosphorylation (Autophos) using 0.5 micrograms of 6X-His ACK1 incubated with gamma-^32^P-ATP for 10 min at 30°C. The reaction was evaluated by SDS-PAGE and autoradiography (*right*). The position of molecular weight standards is shown on the left and 6X-His ACK1 on the right. (B) Identification of cortactin tyrosine residues phosphorylated by ACK1. Purified GST-cortactin murine wild type (WT) or Y421/466/482F (triple tyrosine mutant; TYM) (0.5 micrograms) were incubated in kinase assays without or with 3U of purified Src (*left*) or with the indicated amounts of purified 6X-His ACK1 (*middle* and *right*) in the presence of [gamma-^32^P]ATP. Reactions were separated by SDS-PAGE and analyzed by audioradiography. Arrows indicate the positions of phosphorylated GST-cortactin and 6X-His ACK1 (*left*). (C) Determination of the K*_M_* of ACK1 for cortactin. Graphical representation showing ACK1 phosphorylation kinetics for cortactin calculated from the kinase assay in *B* (*right* panel). Percent of maximum phosphorylation signal measured by densitometry is represented on the ordinate versus concentration of ACK1 (in micrograms) on the abscissa. The calculated K*_M_* for cortactin phosphorylation is shown. Data in B and C are representative from three independent experiments. (D) ACK1 knockdown reduces cortactin phosphorylation on tyrosine 421. 1483 cells transfected with scrambled control (Ctl) siRNA or ACK1-targeting siRNA were lysed and analyzed by Western blotting for ACK1 knockdown (ACK1) and cortactin pY421 phosphorylation (Cort pY421). Beta actin was blotted to verify equivalent protein loading. Blots are representative of two independent experiments.

### Recombinant Protein Production

The ACK1 coding sequence was PCR amplified as an EcoRI/BglII fragment and subcloned into the baculoviral expression vector pAcHLT-B (Pharmingen). Baculovirus production and purification of 6X-His ACK1 was essentially as described [48]. GST-cortactin proteins were produced and purified as before [49]. To produce GST-cortactin Y421/466/482F (triple tyrosine mutant; TYM), the coding sequence of FLAG-cortactin TYM [50] was PCR amplified with flanking BamHI/EcoRI ends and subcloned into pGST-parallel 2 [49].

**Figure 3:**
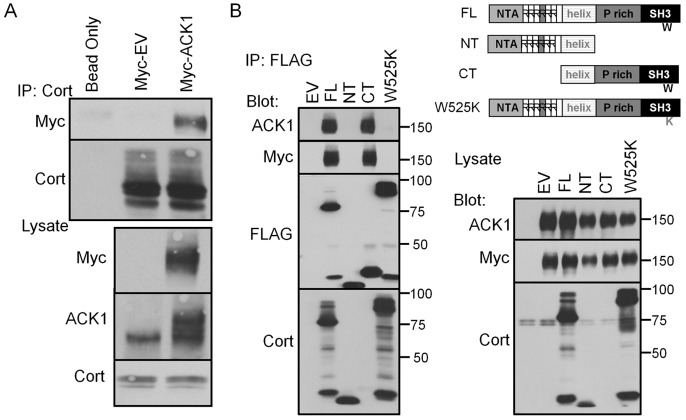
Identification of the ACK1 binding region in cortactin. (A) Endogenous cortactin was immunoprecipitated from 1483 cells transfected with empty Myc vector (EV) or with Myc-tagged ACK1. Non-transfected cells were mock precipitated (Bead Only) as background control. Immune complexes were assayed by Western blotting with anti-Myc and anti-cortactin and antibodies. Total cell lysates from transfected cells were probed with anti-Myc, Anti-ACK1 and anti-cortactin antibodies. (B) Clarified lysates from 293T cells coexpressing Myc-tagged ACK1 with either empty FLAG vector (EV), full-length (FL), N-terminal (NT), C-terminal (CT) or SH3-binding deficient (W525K) FLAG-cortactin constructs (*schematic diagram at top right*) were immunoprecipitated with anti-FLAG antibodies. Membranes were immunoblotted with anti-ACK1, anti-Myc, anti-FLAG and anti-cortactin antibodies (*left*). Expression of recombinant ACK1 and cortactin proteins for each transfection condition were confirmed by Western blotting of total cells lystes with the indicated antibodies (*bottom right*). The positions of molecular weight markers are shown on the right. All blots are representative of ≥ three independent experiments.

### Kinase Assays

Wild type and TYM GST-cortactin proteins were incubated with or without 6X-His ACK1 or Src (Upstate) in 50 millimolar HEPES (pH = 7.0), 5 millimolar MgCl_2_, 5 millimolar MnCl_2_ and 10 microcuries [gamma^32^P]ATP (PerkinElmer) in 50 microliters. Reactions were incubated at 30°C for 10 min and terminated by the addition of hot 2X-SDS sample loading buffer. Reactions were resolved by SDS-PAGE, the gels dried and subjected to autoradiography. Autoradiograms were quantified by ImageJ. Enzyme kinetics for cortactin phosphorylation by ACK1 were calculated using the predicted molecular weight for ACK1 including the 6X-His tag (119.33 kilodaltons) and autoradiogram band intensities to generate a Lineweaver-Burke plot and the subsequent K*_M_* value.

### Antibodies

Anti-ACK1 (clone A-11) and EGFR (clone 1005) were purchased from Santa Cruz. Anti-phosphotyrosine (RC 20) was purchased from BD Transduction. Anti-Cdc42, RhoA, Anti-FLAG (DDDDK tag), anti-Myc epitope tag (4A6) and anti-beta-actin were purchased from EMD Millipore. Anti-cortactin (4F11) and anti-pS418 cortactin were previously described [51], [52]. Anti-pY421 cortactin was from Invitrogen. Anti-HA (3F10) was from Roche.

**Figure 4:**
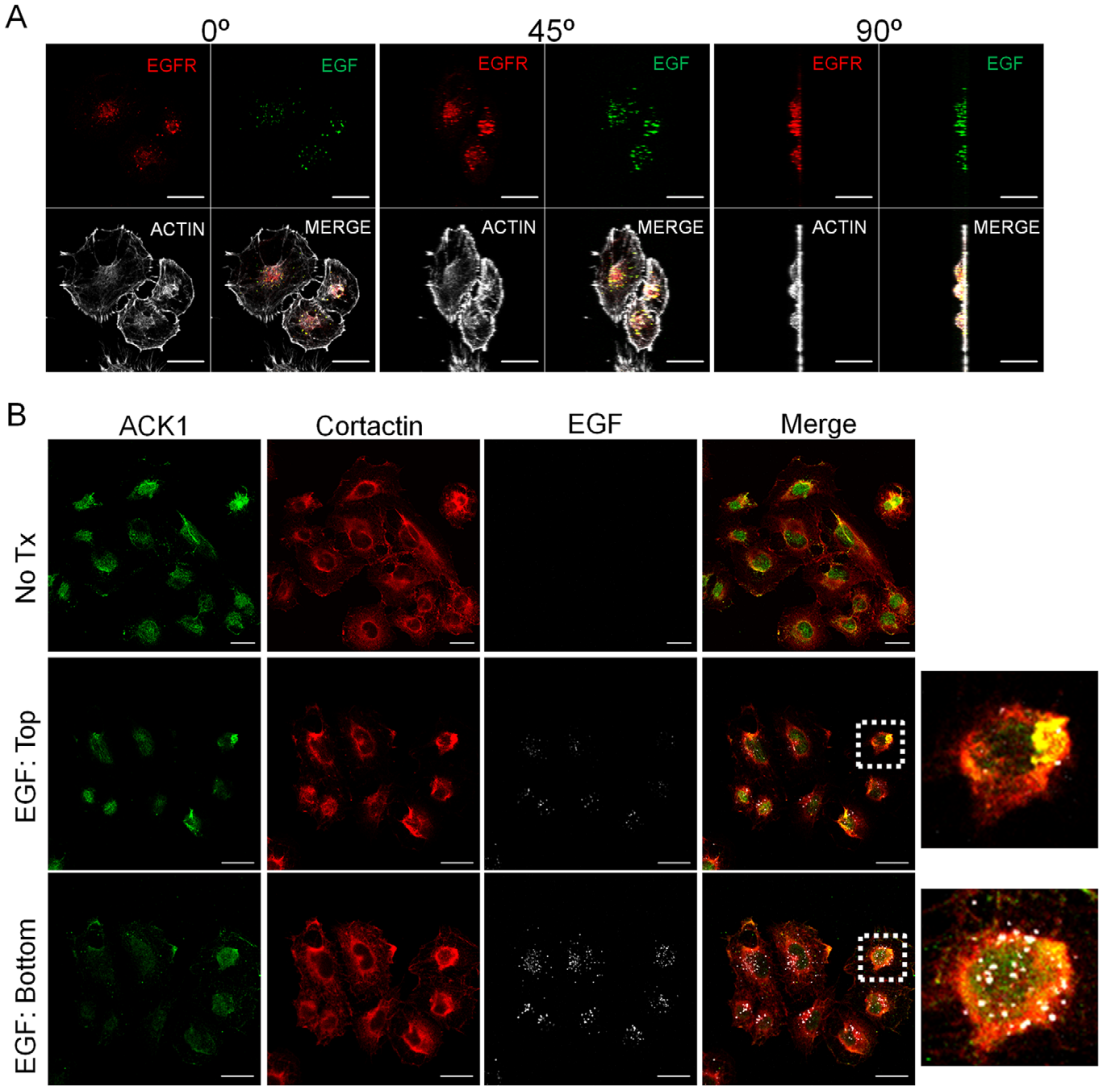
Cortactin localizes with ACK1 in vesicles containing ligand-bound EGFR. (A) 1483 cells serum starved for 16 h were stimulated with 100 nanograms/milliter Alexa Fluor-488 conjugated EGF (green) for 30 min. Cells were fixed and labeled with phalloidin (Actin; pseudocolored white) and anti-EGFR antibodies (pseudocolored red). Cells were evaluated by confocal microscopy and images rotated 45° and 90° as indicated to demonstrate EGF/EGFR colocalization throughout the z-plane. (B) Serum starved (No Tx) 1483 cells were stimulated with FITC-EGF (pseudocolored white) as in (A). Cells were fixed and labeled with anti-ACK1 (green) and cortactin (red) antibodies. Confocal images of labeled EGR in the apical (top) and ventral (bottom) cellular regions are shown. Dashed boxes in the merged images indicate the areas enlarged in the photos to the *right*. Scale bars, 20 micrometers.

### Immunoprecipitation and Western Blotting

For immunoprecipitations, cells were lysed in NP40 buffer (20 millimolar HEPES-KOH, pH 7.8, 50 millimolar KCl, 1 millimolar EDTA and 1% NP40). Five micrograms of primary antibody (anti-cortactin, EGFR, and HA) were incubated with 0.5 milligrams clarified lysates for 2 h at 4°C, then incubated with 40 microliters of Protein A/G beads (Thermo Scientific) for 1 h at 4°C. FLAG immunoprecipitations were preformed with 40 microliters of anti-FLAG M2 affinity gel (Sigma). Immune complexes were collected by centrifugation, washed twice with NP40 Buffer, separated by SDS-PAGE and Western blotted as described [50]. In some cases, cells were serum starved for 16 h and stimulated with 100 nanograms/milliliter EGF (Millipore) prior to immunoprecipitation and/or Western blotting. Band intensities were quantified using ImageJ.

### Confocal Microscopy

Cells were serum-starved for 16 h and stimulated with 100 nanograms/milliter unlabeled EGF (Millipore) or Alexa Fluor-488 conjugated EGF (Invitrogen) for 30 minutes before fixation. Cells were fixed with fresh 4% formaldehyde and permeabilized with 0.4% Triton X-100 in PBS. Primary antibodies listed were diluted with 5% BSA in PBS. Secondary antibodies used were Alexa Fluor 405 goat anti-rabbit and Alexa Fluor 647 goat anti-mouse (Molecular Probes). F-actin was labeled with rhodamine-conjugated phalloidin (Molecular Probes). Cells were mounted in Fluoromount-G (Southern Biotech) and imaged with a Zeiss LSM510 confocal microscope using AIM software (Carl Zeiss MicroImaging). Invadopodia and matrix-degradation assays were preformed as previously described [47].

**Figure 5:**
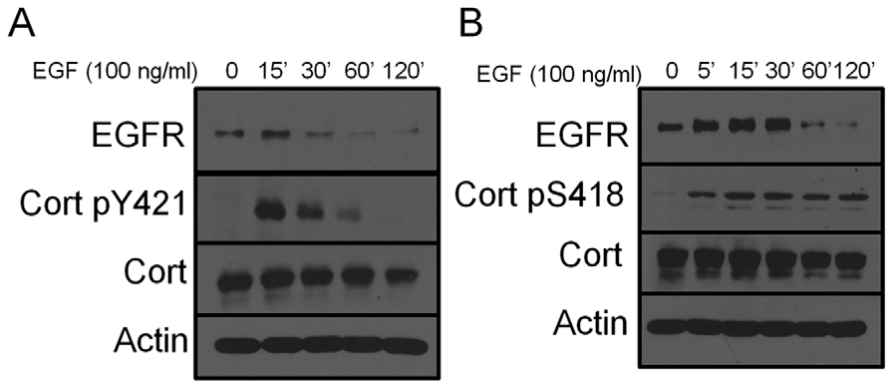
Cortactin tyrosine phosphorylation is downregulated during EGFR degradation. 1483 cells were serum starved for 16 h then stimulated with 100 nanograms/milliter EGF for the indicated times. Cell lysates were analyzed after stimulation by immunoblotting with anti-EGFR, anti-cortactin, anti-actin, and anti-cortactin pY241 (A) or anti-cortactin pS418 antibodies (B). Blots are representative of three independent experiments.

## Results

### Activation of Cdc42 Enhances Cortactin Tyrosine Phosphorylation through ACK1

Our previous work demonstrated that cortactin tyrosine phosphorylation is regulated in part by the small GTPase Rac1 [50]. To determine if other Rho family members influence cortactin tyrosine phosphorylation, we conducted a series of co-transfection experiments utilizing dominant negative and constitutive active constructs encoding the Rac1-related GTPases RhoA and Cdc42 with FLAG-tagged cortactin and assayed total cortactin tyrosine phosphorylation by Western blotting. Expression of RhoA activation variants caused no detectible changes in cortactin tyrosine phosphorylation (Figure S1). However, co-expression of constitutive active Cdc42 (L61) consistently resulted in > six-fold increases in cortactin tyrosine phosphorylation (Figure 1A). Activated Cdc42 is the only small GTPase that directly interacts with the tyrosine kinase ACK1 through a conserved Cdc42/Rac interaction binding (CRIB) motif [53], stimulating ACK1 tyrosine kinase activity [54], [55]. To determine if ACK1 was the kinase potentially responsible for cortactin tyrosine phosphorylation downstream of activated Cdc42, we conducted FLAG-cortactin co-transfection experiments with HA-tagged wild-type (WT) ACK1, kinase null (KR) and Cdc42 binding null (BD) mutant constructs. Analysis of tyrosine phosphorylation status by Western blotting indicated a four-fold increase in cortactin phosphorylation when co-expressed with wild type ACK1. Co-expression of ACK1 KR did not elevate cortactin tyrosine phosphorylation levels, whereas ACK1 BD expression resulted in a 1.8 fold increase (Figure 1B). The level of cortactin tyrosine phosphorylation corresponded with the degree of autophosphorylation present in the assayed ACK1 constructs (Figure 1B), with the low kinase activity of ACK1 BD likely due to Cdc42-independent activation [13]. These data collectively suggest that Cdc42 stimulates cortactin tyrosine phosphorylation through activation of ACK1.

**Figure 6:**
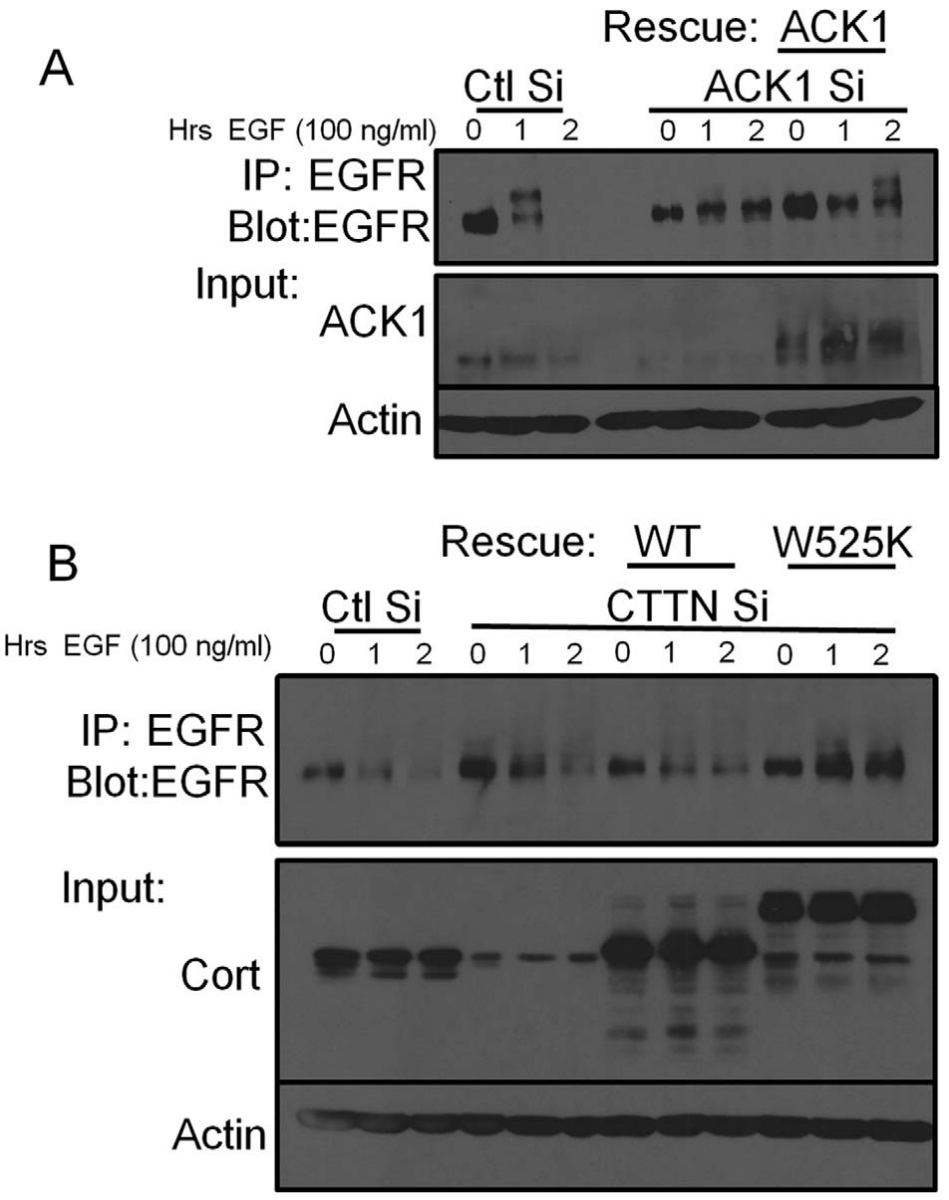
EGFR downregulation requires ACK1 and the cortactin SH3 domain. (A) 1483 cells were transfected with non-targeting (Ctl) or human-specific ACK1 siRNA (ACK1 Si) for 48 h. Murine Myc-ACK1 was subsequently transfected into ACK depleted cells to rescue ACK1 expression. Cells were serum starved for 16 h and then treated with EGF for the indicated times. Following stimulation, clarified lysates were immunoprecipitated and immunoblotted with anti-EGFR antibodies. Total cell lysates were immunoblotted with anti-ACK1 and anti-actin antibodies. (B) 1483 cells were transfected with a non-targeting (Ctl) or cortactin specific siRNA (CTTN Si) for 48 h. Cortactin expression was rescued by transfection with FLAG-cortactin wild type (WT) or with an SH3-null binding mutant (W525K). Cells were serum starved for 16 h prior to EGF stimulation for the indicated times. EGFR was immunoprecipated and immunoblotted with anti-EGFR antibodies. Total cell lysates were immunoblotted with anti-cortactin to verify knockdown and expression of the FLAG-cortactin rescue constructs. Western blotting with anti-actin antibodies was conducted to verify equal protein loading. Blots are representative of two independent experiments.

### ACK1 Directly Phosphorylates Cortactin on Tyrosines 421/466/482

Phosphorylation of cortactin on tyrosines 421, 466 and 482 by several non-receptor tyrosine kinases indicates that these residues serve as important regulatory targets that mediate signals responsible for governing cortical actin-based processes. To determine if ACK1 directly phosphorylates cortactin on these residues, we generated baculovirus encoding full-length 6X-histidine tagged recombinant ACK1 and purified the kinase from infected SF9 cells. Purified ACK1 migrated at a M_r_ of 145 kDa by SDS-PAGE, similar to the mobility observed in other cell systems [14], [16] and possessed functional kinase activity as determined by autoradiographical analysis of autophosphorylation (Figure 2A). Incubation of 6X-His ACK1 with GST-cortactin wild type (WT) in kinase reactions containing [gamma-^32^P]ATP resulted in GST-cortactin phosphorylation similar to levels observed when cortactin was incubated with purified Src (Figure 2B). Incubation of GST-cortactin containing tyrosine to phenylalanine mutations at Y421, Y466 and Y482 (triple tyrosine mutant; TYM) with 6X-His ACK1 nearly abolished cortactin phosphorylation (Figure 2B), with residual phosphorylation of additional sites evident at extended autoradiogram exposure times (data not shown). Increasing amounts of 6X-His ACK1 with fixed amounts of GST-cortactin WT resulted in saturable levels of cortactin phosphorylation (Figure 2B and C), with a calculated Michaelis constant for cortactin phosphorylation equaling ∼140.7 nM. To evaluate the level of endogenous phosphorylation of the identified cortactin tyrosine sites by ACK1 in cells, 1483 HNSCC cells were transfected with non-targeting (Ctl) or ACK1-targeted small interfering (si)RNA. Immunoblot analysis indicated an 82% knockdown of ACK1 expression, which corresponded with an 84% decrease in cortactin phosphorylation at tyrosine 421 (Figure 2D). These results collectively indicate that ACK1 directly phosphorylates cortactin primarily at Y421/Y466/Y482 in a manner similar to other cortactin-targeting tyrosine kinases.

### The Cortactin SH3 Domain Mediates Binding to ACK1

In order to determine how cortactin interacts with ACK1, 1483 HNSCC cells were transfected with Myc-tagged ACK1 (to enhance ACK1 detection) and assayed for cortactin binding by co-immunoprecipitation. Myc-ACK1 precipitated with endogenous cortactin (Figure 3A), suggesting the two proteins interact. The immunoprecipitation was specific, since beads lacking anti-cortactin antibody did not precipitate Myc-ACK1 (Figure S2). We next cotransfected Myc-ACK1 with FLAG-cortactin deletion constructs in order to map the cortactin subdomian(s) responsible for ACK1 binding. Myc-ACK1 precipitated with FLAG-cortactin full length (FL) and carboxyl terminal (CT) (amino acids 350–546) proteins, but not with a FLAG-cortactin protein containing the amino terminal (NT) Arp2/3 and F-actin binding regions (Figure 3B). These results localize the cortactin binding site for ACK1 to the carboxyl-terminal half of cortactin. The cortactin SH3 domain at the carboxyl-terminus binds to proline-rich segments in several proteins involved in actin regulation and endocytosis [56] while ACK1 contains a several putative proline-rich segments within its carboxyl terminus [53]. To determine if the cortactin SH3 domain binds to ACK1, Myc-ACK1 was co-transfected with a cortactin construct containing a point mutation within the SH3 domain that ablates binding to proline-rich ligands (W525K) [46]. This protein displays retarded electrophoretic mobility in SDS-PAGE, presumably due to altered conformation introduced by the point mutation as previously observed [46]. FLAG-cortactin W525K failed to co-precipitate Myc-ACK1 (Figure 3B), indicating that cortactin SH3 domain is responsible for linking cortactin to one or more proline rich sequences in ACK1.

### ACK1 and Cortactin Localize in Vesicles Containing Ligand-bound EGFR

Since ACK1 and cortactin have been individually shown to participate in regulating EGFR surface expression following ligand stimulation [15], [18], [40], we determined if both proteins localized at sites containing activated EGFR. Initial experiments in 1483 cells stimulated with Alexa Fluor-488 conjugated EGF for 30 min indicated that EGF/EGFR complexes were present throughout the vertical dimension of the cell when analyzed by whole cell confocal analysis, demonstrating internalization of activated EGFR in this system (Figure 4A). Confocal imaging of the apical and basal planes of fluorescently-labeled EGF stimulated 1483 cells co-immunostained with antibodies against ACK1 and cortactin indicated co-localization of ACK1 and cortactin at apical internalization regions as well as in EGF-containing vesicles at the ventral surface of the cell (Figure 4B). These vesicles contained activated EGFR that co-localized with cortactin (Figure S3), collectively demonstrating that cortactin localizes with ACK1-containing vesicles associated with activated EGFR during receptor internalization.

In addition to regulating CME, cortactin tyrosine phosphorylation has a prominent role in governing podosome and invadopodia function in invasive cells [47], [57], [58]. Myc-ACK1 did not localize podosomes or invadopodia in invasive Src-transformed HNSCC cells, nor did knockdown of ACK1 expression impact the ability of podosomes/invadopodia to degrade ECM (Figure S4). These results suggest that ACK1 does not participate in cortactin phosphotyrosine-based signaling involved in regulating podosome or invadopodia function.

### Cortactin Phosphorylation is Differentially Regulated during EGFR Degradation

The ability of ACK1 to bind and phosphorylate cortactin, along with the localization of ACK1 and cortactin with EGFR during ligand stimulated receptor internalization, suggests that cortactin tyrosine phosphorylation may be modulated during the internalization process. Additionally, EGFR activation stimulates ERK1/2 activity, which in turn phosphorylates cortactin at S405 and S418 to regulate cortactin SH3 domain binding to N-WASp and other proteins [59], [60]. To monitor cortactin tyrosine and serine phosphorylation status during EGFR internalization, Western blotting of whole cell lysates was conducted with phosphorylation-specific antibodies against tyrosine 421 (pY421) and serine 418 (pS418). EGF treatment of starved 1483 cells induced cortactin tyrosine phosphorylation evident at 15 min, which gradually decreased until it was not detectable two h post stimulation (Figure 5A). The reduction of cortactin tyrosine phosphorylation mirrored ligand-induced EGFR downregulation and was not due to decreased cortactin protein levels. EGF-induced ERK1/2 cortactin phosphorylation persisted throughout the entire time course and did not decrease during EGFR degradation (Figure 5B). These results indicate that cortactin tyrosine and serine phosphorylation undergoes disparate regulation during EGFR downregulation.

### The Cortactin SH3 Domain is Required for Ligand-induced EGFR Downregulation

To determine if a potential ACK1/cortactin linkage contributes to EGFR internalization, we conducted siRNA knockdown and rescue experiments of ACK1 and cortactin to evaluate effects on EGFR degradation. 1483 cells treated with scrambled (Ctl Si) siRNA demonstrated complete EGFR degradation at two h when evaluated by immunoprecipation (Figure 6A). RNAi depletion of ACK1 (ACK1 Si) prevented EGFR downregulation in ligand stimulated cells, indicating that ACK1 plays an essential role in promoting EGFR internalization in 1483 cells. Rescue of ACK1 Si cells by expression of Myc-ACK1 restored the ability of 1483 cells to degrade EGFR, albeit with delayed degradation kinetics (Figure 6A).

Finally, to determine if cortactin plays a similar role to ACK1 in EGFR downregulation, EGFR degradation was assayed in cortactin knockdown and rescued 1483 cells following EGF treatment. Transfection of cortactin siRNA (CTTN Si) resulted in increased EGFR accumulation in starved cells and slowed EGFR degradation compared with control siRNA treatment (Figure 6B). Tranfection of FLAG-cortactin in CTTN Si cells also delayed EGFR degradation compared to control siRNA cells (Figure 6A). This result may be due to the inhibitory effects of cortactin overexpression as previously reported in HNSCC cells [40]. However, rescue of CTTN Si cells with FLAG-cortactin W525K blocked EGFR degradation in a manner similar to that in stimulated cells with ACK1 knockdown (Figure 6A and B). Overall, this indicates that ACK1 and the cortactin SH3 domain are required for ligand induced EGFR downregulation, potentially through a direct interaction between the two molecules.

## Discussion

ACK1 has been implicated in multiple aspects of normal cellular and neoplastic processes, including growth, invasion and endocytic regulation of growth factor receptors [13]. Ligand induced downregulation of EGFR is an important regulatory mechanism for controlling EGFR signaling that involves CME of the receptor and ultimate trafficking to lysosomes [2], [61]. Evidence to date indicates that regulated actin polymerization at all points of the internalization pathway is vital in driving the membrane dynamics necessary for EGFR internalization, with cortactin serving as an important scaffold by coupling Arp2/3 activity to cytoplasmic membrane surfaces during internalization [28], [34], [62]. Our present findings provide evidence that ACK1 directly phosphorylates cortactin at tyrosine sites that provide enhanced Arp2/3-based actin polymerization, which may be utilized to facilitate EGFR internalization. ACK1 associates with the cortactin SH3 domain, providing a potentially important interaction point in promoting receptor internalization to regulate EGFR surface expression.

Our data indicating that Cdc42 stimulates direct ACK1 phosphorylation of cortactin is in agreement with the localization of these proteins at clathrin coated pits during early endocytic stages, supporting cortactin as an ACK1 substrate during receptor internalization [17], [29], [63]. We show that ACK1 phosphorylates the canonical cortactin tyrosine residues 421/466/482 that are also targeted by Src [64], Abl/Arg [36], Fer [65], c-Met [66], Fyn [67] and HER2 [68]. Src activation triggered by EGFR contributes to receptor endocytosis and is responsible for simulating ACK1 activity [69], [70]. Src phosphorylation of cortactin is required for transferrin receptor CME [33], [34], indicating that cortactin is also targeted by Src during endocytosis. In addition, Abl is activated during EGFR stimulation but plays a negative role in receptor downregulation [12]. The resultant aggregate activity of multiple kinases towards the same cortactin phosphorylation sites during EGFR endocytosis increases the level of complexity regarding regulation of endocytic events controlled by cortactin tyrosine phosphorylation. Nevertheless, our direct phosphorylation data with purified proteins combined with localization of ACK1 and cortactin with activated EGFR suggests that at least a subset of cortactin is likely phosphorylated by ACK1 during receptor internalization. The additional low level ACK1 phosphorylation present in cortactin lacking tyrosines 421/466/482 may be due to targeting of other tyrosine residues phosphorylated downstream of EGFR [71] or possible phosphorylation of serine/threonine sites due dual specificity of ACK1 [72]. Identification of these additional cortactin phosphorylation sites targeted by ACK1 remains to be determined.

Deletion mapping and point mutational studies indicate that the cortactin SH3 domain mediates binding to ACK1. Analysis of the ACK1 primary sequence by the STRING database (http://string.ebml.de) indicates that the ACK1 proline rich region contains a region between amino acids 735–743 (KPQVPPRVP) that is a partial consensus with proline rich regions in other proteins that bind the cortactin SH3 domain [73]. We are currently working on identifying the precise cortactin binding site in ACK1 within the proline rich region. In addition to ACK1, the cortactin SH3 domain binds to proline rich sequences in CD2AP [39] and dynamin2 [29] during CME. The multiplex binding of the cortactin SH3 domain to proteins with different functions likely delineates specific roles during endocytic receptor internalization. Cortactin SH3 domain binding to CD2AP links Arp2/3 actin nucleation to EGFR during internalization through a CD2AP complex containing Cbl and endophilin [39]. Endophilin contains an N-BAR (Bin–amphiphysin–Rvs) domain that inserts into the cytoplasmic plasma membrane face, inducing membrane curvature to initiate vesicle formation [74]. The interaction of cortactin with dynamin2 has been shown to regulate fission of clathrin coated vesicles through Src phosphorylation of cortactin and concurrent regulation of actin dynamics [28], [32], [34]. Cortactin binding to ACK1 likely occurs later in the internalization pathway, since ACK1 localizes at the base of exaggerated elongated tubular invaginations reminiscent of endosomes present in dynamin-null cells [17] as well as on early endosomes in other cell types [15]. The sequential binding of cortactin SH3 domain proteins is supported by a comprehensive analysis of CME using live cell imaging, where peak recruitment of dynamin2 occurred before ACK1 to the preassociated cortactin-containing F-actin network [75]. However, dynamin2 has recently been shown to also regulate late stages of endosomal budding [76], suggesting overlapping and redundant functions of cortactin SH3 domain binding proteins in receptor internalization and trafficking.

The findings that ACK1 and the cortactin SH3 domain are required for EGFR degradation point to a functional role for this complex in regulating EGFR surface expression. The loss of cortactin tyrosine phosphorylation during receptor downregulation (Figure 5A) suggests that ACK1-targeted cortactin tyrosine residues are dephosphorylated while ACK1 is still bound to cortactin. EGFR-containing late endosomes are targeted to MVBs, where dephosphorylation of tyrosine residues in the EGFR cytoplasmic domain by the ER-associated protein tyrosine phosphatase PTP1B triggers subsequent lysosomal targeting and degradation [77]. Fusion of late endosomes containing phosphorylated cortactin and ACK with MVBs would place cortactin in close proximity to PTP1B, which dephosphorylates cortactin [71]. Dephosphorylated cortactin would be released from the vesicle surface and recycled with ACK1, or ACK1 may remain constitutively bound to and degraded with EGFR [21], [23]. While dephosphorylation of cortactin would remove Nck1 docking sites required for the assembly of N-WASp/WIP Arp2/3 nucleation complexes, cortactin remains phosphorylated on the ERK1/2 phosphorylation sites important for maintaining the SH3 domain in an “open” conformation [52], [60]. This would to allow binding to N-WASp and presumably other SH3 domain binding proteins, providing for and maintaining Arp2/3-based actin polymerization during vesicle trafficking [78].

In summary, we have demonstrated that cortactin is an ACK1 substrate that potentially links the ACK1 EGFR internalization pathway to Arp2/3-based cortical actin regulation. It is likely that this pathway plays important roles in tumor progression, since elevated expression of ACK1 and cortactin due to gene amplification is found in several tumor types [79], [80], and dysregulation of ACK1 and cortactin expression alters EGFR internalization dynamics [18], [40]. How cortactin tyrosine phosphorylation is regulated by ACK1 and other cortactin kinases during internalization, as well as an improved understanding how the binding of cortactin SH3 domain-associated proteins are orchestrated are important questions for future consideration.

## Supporting Information

Figure S1 **RhoA activation does not impact cortactin tyrosine phosphorylation.** COS1 cells were cotransfected with FLAG-cortactin WT and Myc-tagged RhoA constructs as indicated. EV; empty vector. Cells were lysed 18 h after transfection, FLAG-cortactin immunoprecipitated and total cortactin tyrosine phosphorylation analyzed by Western blotting with a pan anti-phosphotyrosine antibody (pTyr). The blot was then stripped and reprobed for total cortactin levels (Cort). Equal amounts of total cell lysates were blotted in parallel with an anti-RhoA monoclonal antibody to confirm expression of recombinant RhoA forms (present at a higher M*r* than endogenous RhoA in the respective transfected lanes). Ratios of tyrosine phosphorylated (pTyr) cortactin to total cortactin levels are indicated at the bottom. Blots are representative from three independent experiments.
(TIF)

Figure S2 **Specificity of the Myc-ACK1-cortactin co-immunoprecipitation.** 293T cells transiently transfected with Myc-ACK1 were lysed and immunoprecipated with Protein A/G beads alone (Beads Only) or with Protein A/G beads bound to anti-cortactin antibody (Anti-CTTN). Immune complexes were resolved by SDS-PAGE and blotted for ACK1 and cortactin (Cort) as indicated. A non-specific binding product (NS) is shown as a control for equal loading. Blots are representative of two independent experiments.
(TIF)

Figure S3 **Cortactin localizes with vesicles containing activated EGFR.** 1483 cells were serum starved for 16 h and then left either untreated (No Tx) or stimulated with AlexaFlour-488 EGF (100 nanograms/milliliter, green) for 30 min before fixation. Cells were stained with anti-EGFR (red) and anti-cortactin (blue) antibodies. Confocal images of labeled EGR in the apical (top) and ventral (bottom) cellular regions are shown. Scale bars, 20 micrometers.
(TIF)

Figure S4 **ACK1 is not a component of cortactin-containing invasive subcellular structures.** (A) 584 cells cotransfected with activated Src kinase (527F) and Myc-ACK1 were plated on coverslips, fixed and labeled with with rhodamine phalloidin (Actin), anti-Myc (blue) and anti-cortactin (green) antibodies. Src-induced podosome rosettes (left panels) are identified as yellow circular aggregates; individual invadopodia (right panels) as subnuclear ventral puncta in the merged actin/cortactin images (white arrows). Arrowheads denote actin/cortactin containing lamellipodia. (B) 1483 or 584 cells were transfected with non-targeting siRNA (Ctl) or siRNA targeting ACK (Si) and analyzed by Western blotting with anti-ACK1 and anti-actin antibodies. (C) 584 cells expressing activated Src were plated on FITC-gelatin coated coverslips (pseudocolored white) for 24 h, fixed and labeled with rhodamine phalloidin (Actin; red) and anti-cortactin (green) antibodies. Scale bar, 20 micrometers.
(TIF)
