# Supplementary figures and images for: Cortactin Is a Substrate of Activated Cdc42-Associated Kinase 1 (ACK1) during Ligand-induced Epidermal Growth Factor Receptor Downregulation
Source: PLoS One. 2012 Aug 30;7(8):e44363. doi: 10.1371/journal.pone.0044363 (PMC3431376; doi:10.1371/journal.pone.0044363)

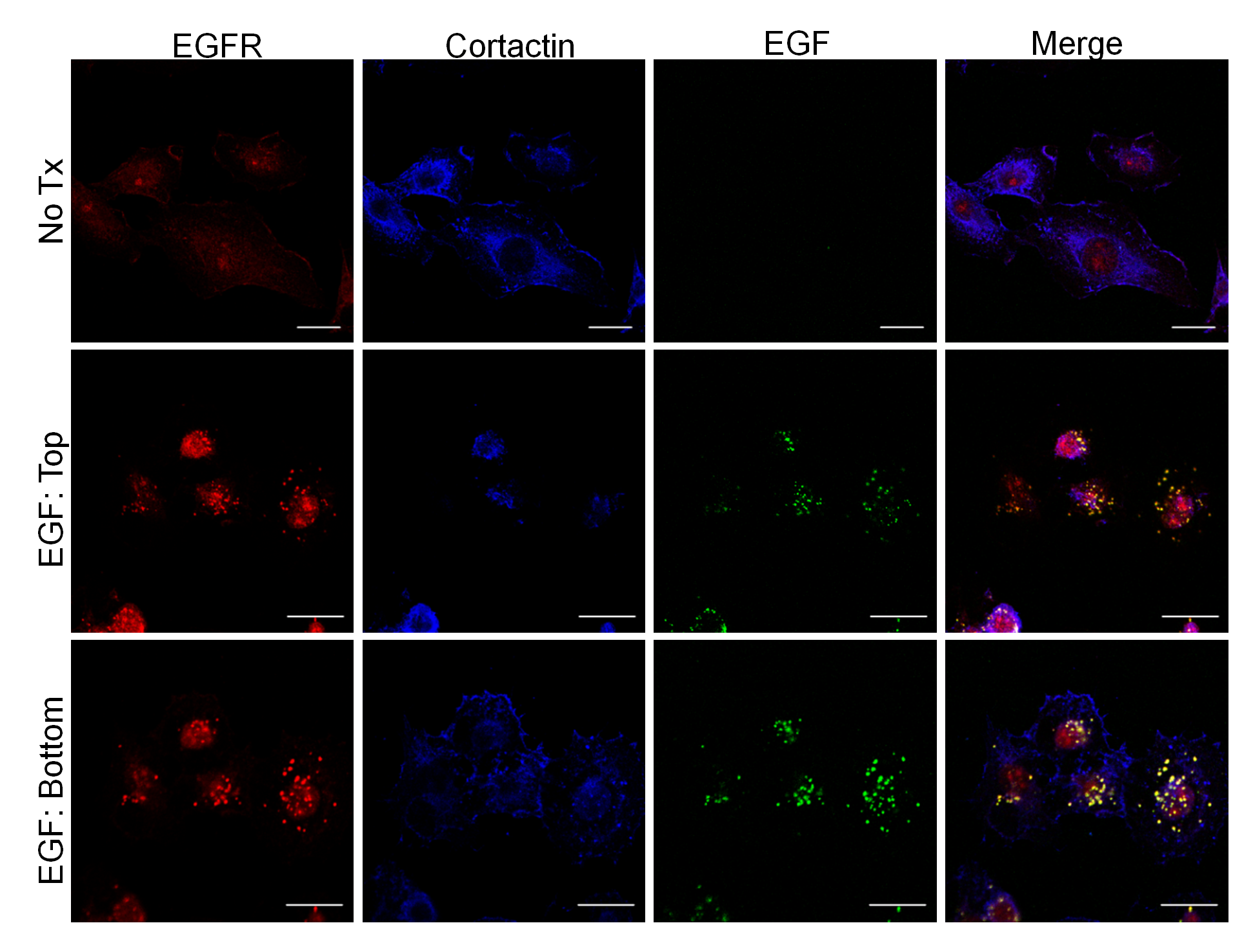

Supplement: Figure S3 — Cortactin localizes with vesicles containing activated EGFR. 1483 cells were serum starved for 16 h and then left either untreated (No Tx) or stimulated with AlexaFlour-488 EGF (100 nanograms/milliliter, green) for 30 min before fixation. Cells were stained with anti-EGFR (red) and anti-cortactin (blue) antibodies. Confocal images of labeled EGR in the apical (top) and ventral (bottom) cellular regions are shown. Scale bars, 20 micrometers. (TIF) [file pone.0044363.s003.tif]

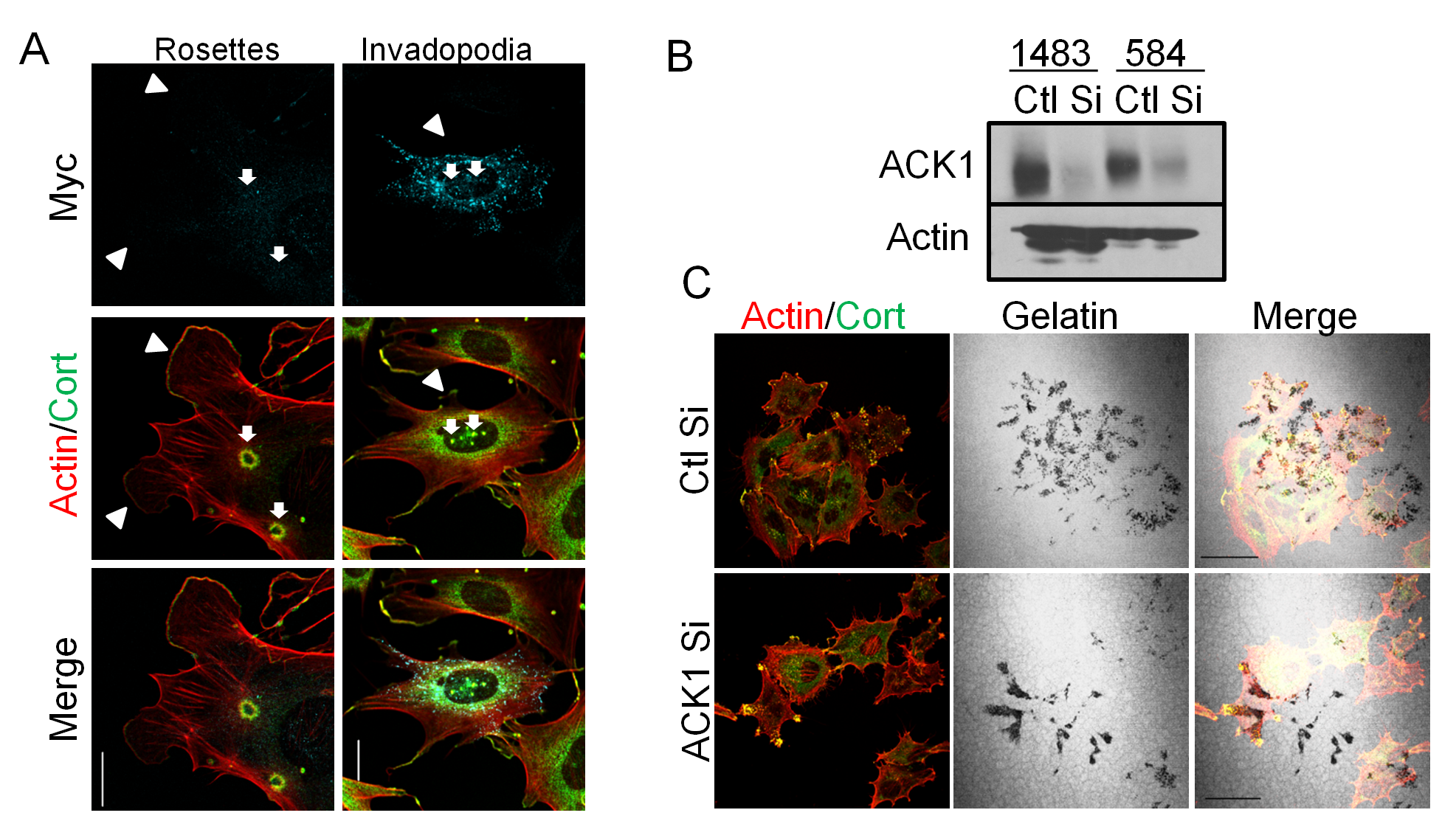

Supplement: Figure S4 — ACK1 is not a component of cortactin-containing invasive subcellular structures. (A) 584 cells cotransfected with activated Src kinase (527F) and Myc-ACK1 were plated on coverslips, fixed and labeled with with rhodamine phalloidin (Actin), anti-Myc (blue) and anti-cortactin (green) antibodies. Src-induced podosome rosettes (left panels) are identified as yellow circular aggregates; individual invadopodia (right panels) as subnuclear ventral puncta in the merged actin/cortactin images (white arrows). Arrowheads denote actin/cortactin containing lamellipodia. (B) 1483 or 584 cells were transfected with non-targeting siRNA (Ctl) or siRNA targeting ACK (Si) and analyzed by Western blotting with anti-ACK1 and anti-actin antibodies. (C) 584 cells expressing activated Src were plated on FITC-gelatin coated coverslips (pseudocolored white) for 24 h, fixed and labeled with rhodamine phalloidin (Actin; red) and anti-cortactin (green) antibodies. Scale bar, 20 micrometers. (TIF) [file pone.0044363.s004.tif]
